# Screening and Management of Obstructive Sleep Apnea and Daytime Sleepiness Among Professional Drivers in Tunisia: Protocol for a Machine Learning Study

**DOI:** 10.2196/70441

**Published:** 2025-08-15

**Authors:** Sameh Msaad, Nesrine Kammoun, Rahma Gargouri, Rim Khemakhem, Amira Triki, Narjes Abid, Sonia Fehri, Kaouthar Kallel, Rim Kammoun, Leila Douik EL Gharbi, Sonia MaalejBellaj, Heni Bouhamed, Ahmed Abdelghani, Chiraz Aichaouia, Mohamed Turki, Samy Kammoun

**Affiliations:** 1 Faculty of Medicine of Sfax University of Sfax Sfax Tunisia; 2 Department of Respiratory and Sleep Medicine University Hospital Hedi Chaker of Sfax Sfax Tunisia; 3 Faculty of Medicine of Tunis Tunis El Manar University Tunis Tunisia; 4 Tunisian Occupational Health and Safety Institute Tunis Tunisia; 5 Department of Pulmonology Mohamed Taher Maamouri Hospital Nabeul Tunisia; 6 General Administration of Inspection of Occupational Medicine and Occupational Safety Tunis Tunisia; 7 Department of Physiology Habib Bourguiba Hospital Sfax Tunisia; 8 Department of Pulmonology University Hospital of Abderrahmen Mami Aryanah Tunisia; 9 Advanced Technologies for Image and Signal Processing Unit (ATISP) Sfax University Sfax Tunisia; 10 Faculty of Medicine of Sousse University of Sousse Sousse Tunisia; 11 Department of Pulmonology Farhat Hashed Hospital Sousse Tunisia; 12 Military Hospital of Tunis Tunis Tunisia; 13 Private Medical Office of Mohamed Turki Tunis Tunisia

**Keywords:** professional drivers, Tunisia, obstructive sleep apnea, daytime sleepiness, drowsy driving, work productivity, epidemiology, machine learning, deep learning, neural network

## Abstract

**Background:**

Obstructive sleep apnea (OSA) is highly prevalent among professional drivers; however, its true burden in this population remains underexplored and likely underdiagnosed.

**Objective:**

This study aims to determine the prevalence of OSA and excessive daytime sleepiness (EDS) and identify their risk factors among a large representative sample of professional drivers in Tunisia. We will also evaluate the risk of accidents associated with OSA and EDS before and after the treatment.

**Methods:**

This will be a population-based and prospective study of about 3000 professional drivers. Participants will receive a structured questionnaire to evaluate five main outcomes: the likelihood of OSA, EDS, drowsy driving, related sleepiness near misses and accidents, as well as work productivity. Validated self-report measures will be used to evaluate these outcomes. Participants suspected of having OSA or EDS will undergo sleep laboratory investigations, including a sleep study. Participants who have moderate-to-severe OSA will be recommended continuous positive airway pressure (CPAP) treatment. After one year of follow-up, all participants will be re-evaluated with self-report questionnaires. For those treated with CPAP, they will undergo the Maintenance of Wakefulness Test (MWT). We will evaluate several widely used machine learning models in medical diagnosis that are known for their high accuracy, including random forests, extreme gradient boosting, and deep neural networks, to predict the probability of OSA and its association with road traffic accidents.

**Results:**

A total of 127 male drivers participated in the study, with a mean age of 39.22 (SD 8.62) years. Most participants (76/127, 60.3%) had completed secondary education, 54.3% (69/127) were smokers, and the median BMI was 25.6 kg/m^2^. A medical history was reported by 20.5% (26/127) of patients. The median driver experience was 8 (IQR 4.0-15.0) years. Among the drivers, 49 (39.5%) were working night shifts, and 30 (23.6%) were in hazardous materials transportation. Machinery (41.3%) was the most common mode of transportation, followed by trucks (34.9%), and light vehicles (22.2%). Notably, 11 drowsiness accidents were avoided. The overall score of presenteeism was 12.25 out of 100, whereas absenteeism was 1.74 out of 100. The overall daily activity impairment and productivity loss were 11.46 out of 100 and 12.76 out of 100, respectively. Overall, 30 (23.62%) cases were identified with either a pathological Epworth Sleepiness Scale or a positive Berlin score.

**Conclusions:**

Our preliminary findings revealed that a significant proportion of drivers were at a high risk of OSA. Our results will pave the way for the creation of a clinical screening instrument that can identify sleep-wake disturbances in professional drivers. This is likely to have a significant impact on the legal regulations concerning driving fitness and road safety.

## Introduction

Obstructive sleep apnea (OSA) is a chronic breathing sleep disease characterized by recurrent episodes of complete (apnea) or partial (hypopnea) upper airway collapse during sleep, which results in repetitive pauses in breathing, brain arousals, sleep fragmentation, intermittent hypoxemia, and increased sympathetic nervous system activity. OSA is highly prevalent worldwide, with almost 1 billion adults (30-69 y) affected, with prevalence rates ranging from 9.3% to 77.2%, depending on the country [1]. Untreated OSA is associated with adverse health outcomes, including increased cardiovascular disease, metabolic diseases, impaired quality of life, mood disorders, and excessive daytime sleepiness (EDS) [2].

Professional drivers, including bus, taxi, and long-haul truck drivers, are an understudied and medically underserved population, despite their high rates of health issues. Compared to the general population and other occupations, professional drivers often have irregular sleep patterns and tend to smoke and drink more but exercise less, which predisposes them to several comorbidities such as obesity, cardiovascular diseases, cerebrovascular diseases, diabetes, musculoskeletal disorders, mental health disorders, uncorrected visual defects as well as sleep and vigilance disturbances. This increased morbidity seems to be associated with an increased likelihood of accidents and fatalities on the road [3].

In previous studies, professional drivers had been found to have high prevalence rates of OSA, ranging from 28% to 78% [4,5], likely because they tend to be obese, male, and middle-aged adults, the 3 most common risk factors for OSA. People with untreated OSA often experience daytime sleepiness, impaired psychometric vigilance, and do worse in driving simulator studies. Therefore, they are 2-3 times more likely to be involved in traffic accidents in comparison with those without OSA. Despite its heavy disease burden, medical awareness as well as appropriate screening and sleep study referrals of OSA in professional drivers are still lacking, leading to underdiagnosis and undertreatment.

There are about 67503 professional drivers currently employed in Tunisia. Most are men (93.8%), with an average age range of 40-49 years old [6]. According to the National Road Safety Observatory (Observatoire National de la Sécurité Routière), Tunisian roads registered 5796 accidents over 2023 with 1216 deaths [7], most caused by road transport professionals: heavy goods vehicle drivers, bus drivers, collective taxis, and cab drivers. Besides speeding, drunk driving, cell phone use and nonuse of seatbelts, distraction, and drowsiness behind the wheel were identified among the main causes of road accidents. Despite all these alarming statistics, to our knowledge, there are no studies available that have performed large-scale analyses on sleep and vigilance disturbance among Tunisian professional drivers.

The primary objective of this study is to determine the prevalence of OSA and EDS and their risk factors among a large representative sample of Tunisian professional drivers. The secondary objective is to evaluate the accidental risk associated with OSA and EDS before and after treatment. In addition, we aim to develop a reliable screening tool for detecting sleep-wake patterns among professional drivers.

## Methods

### Study Design and Settings

This is a population-based prospective cohort study that will be conducted by the Department of Respiratory and Sleep Medicine at Hedi Chaker University Hospital of Sfax, Tunisia, in collaboration with the Tunisian Occupational Health and Safety Institute. Data collection will be performed from May 1, 2024, to July 31, 2025.

### Population

#### Participants’ Eligibility Criteria

All professional drivers in the private sector, including light vehicle drivers, bus drivers, truck drivers, and bulldozer drivers, who have been working regularly for at least 1 year in any of the 24 provinces of Tunisia, are eligible to participate in the study. Public sector drivers are excluded from the study due to challenges in accessing this demographic in Tunisia, such as bureaucratic obstacles and lengthy approval processes required for research.

#### Noninclusion Criteria

Drivers of the public sector and those who refuse to participate in the study will not be included.

#### Sample Size

To ensure accurate and representative data, we estimated the required sample size using the following equation [8] as 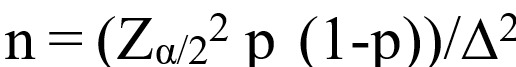. “*Z****_α/2_***” (=2.33) was the normal deviate for a 1-tailed hypothesis at a 1% level of significance; (*“P*=.28”) was the prevalence of OSA reported in a previous study involving 406 professional drivers [4], “***∆***” represents the precision which is arbitrarily fixed at 6%. Using the above formula, the estimated sample size was 3000 professional drivers.

#### Data Collection

Data collection will be performed using a 10-minute face-to-face questionnaire designed in literary Arabic. The questionnaire is introduced by a brief description including information about the study’s purpose and authors, a statement on confidentiality, and specific instructions for each question if needed. It’s composed of a series of forced-choice questions that cover 6 main topics: sociodemographic data, habits, health status and morbidities, and a series of validated self-report measures related to OSA, ESD, and work productivity. At the end of the questionnaire, 2 open-ended questions were added to collect any comments or further information that participants would share with researchers. To assess the reliability and validity of the questionnaire, a pilot test was performed with 40 volunteers before the implementation of the full survey.

### Measures

#### Sociodemographic Data

Demographic data will be collected, including age, gender, weight (kg), height (cm), residence type (rural or urban areas), educational level, and marital status. We will also gather information about the occupational characteristics of the participants, such as seniority at work, engagement in night work, categories of vehicles, average hours per “day” or “week,” and average miles driven per “day” or “week.”

#### Habits and Morbidities

We will collect data on tobacco smoking, alcohol use, hypnotic consumption, and prevalent morbidities such as otolaryngologic, respiratory, cardiovascular, metabolic, autoimmune, and psychiatric diseases, as well as SARS-CoV-2 infection. Participants will also be asked if they were previously investigated or treated for any breathing sleep disorder (snoring, OSA, etc).

#### OSA Evaluation

**Figure 1:**
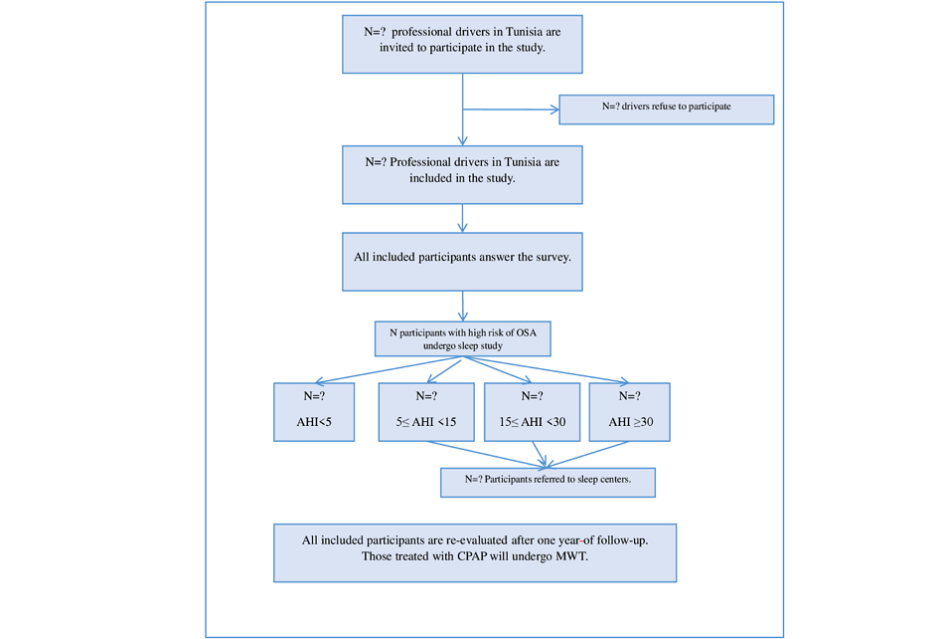
Flow diagram of the study population. AHI: Apnea-Hypopnea Index; CPAP: continuous positive airway pressure, MWT: Maintenance of Wakefulness Test, OSA: obstructive sleep apnea.

Participants who do not have a pre-existing diagnosis of OSA will be evaluated using the validated Arabic version of the Berlin questionnaire (Cronbach α=0.92; Multimedia Appendix 1) [9]. This is a self-administered questionnaire designed to identify participants at increased risk for OSA. It consists of 10 questions that assess three categories of OSA symptoms and signs: (1) snoring and its intensity, frequency and inconvenience to others, as well as witnessed apneas during sleep; (2) EDS and its severity; and (3) history of arterial hypertension or obesity, as defined by a BMI 30 kg/m^2^. In category 1, a positive score is defined as persistent symptoms (3-4 times/wk) in at least 2 questions about snoring. In category 2, a positive score is defined as persistent (3-4 times/wk) EDS, drowsiness behind the wheel, or both. In category 3, a positive score is defined as a history of arterial hypertension or a BMI 30 kg/m^2^. Participants exhibiting more than one positive category are identified at an increased risk for OSA [10] (Figure 1).

#### Daytime Sleepiness

All the participants will be asked to answer a validated Arabic version of the Epworth sleepiness scale (ESS; Cronbach α=0.86; Multimedia Appendix 2) [11]. This is a self-reported questionnaire, which is shown to provide a subjective assessment of the severity of daytime sleepiness over the last 3 months. The ESS is based on a list of questions referring to 8 daily life situations in which the participants rated their tendency to doze off or fall asleep on a scale of 0 (no chance of dozing) to 3 (high chance of dozing). The total ESS is the sum of responses to all individual 8 items and ranges from 0 to 24, with a higher score indicating a higher level of daytime sleepiness. In this study, an ESS score ≥11 is considered suggestive of EDS, while an ESS score ≥16 indicates severe EDS [12].

Drowsy driving is investigated by the following questions [13]: “Have you ever dozed off or fallen asleep while driving.” Each response is rated based on the frequency of drowsy driving episodes: “almost every day,” “1 to 2 times per week,” “1 to 2 times per month,” less than “1 time per month,” or “never or almost never.” For statistical analysis, participants are categorized into 2 groups based on how frequently they experience drowsy driving: those who drive drowsy at least once a month and those who do so less than once a month.

The occurrence of sleep-induced vehicular accidents and near-misses in the last year is recorded by the following question: “Have you ever had or avoided an accident due to drowsiness while driving?” A near-miss accident is defined as an event with a limited impact that could cause an accident (eg, drifting from the lane or crossing a white line) but did not result in any damage. An accident is defined as an event that results in physical injury or property damage.

#### Work Productivity

We will use an Arabic-validated edition of the Work Productivity and Activity Impairment Questionnaire: General Health version 2.2 (WPAI: GH 2.0; Multimedia Appendix 3) to measure how much the general health and symptoms severity affected work productivity and regular activities during the past week.

The WPAI-GH 2.2 is a set of six questions as follows: (1) Currently employed? (2) Number of hours missed from work due to health problems in the past week? (3) Number of hours missed from work due to other reasons in the past week? (4) Number of hours worked in the past week? (5) Degree to which health affected productivity while working (using a 0-10 Visual Analogue Scale)? (6) Degree to which health affected productivity in regular unpaid activities (using a 0-10 Visual Analogue Scale)?

Four main outcomes are generated from the WPAI: GH 2.0 and presented as percentages by multiplying the following scores by 100:

1. Percent of work time missed due to health=Q2/(Q2+Q4) for those who were currently employed.
2. Percent impairment while working due to health=Q5/10 for those who were currently employed and worked in the past week.
3. Percent overall work impairment due to health=Q2/(Q2+Q4) + ((1–Q2/(Q2+Q4)) × (Q5/10)) for those who were currently employed.
4. Percent activity impairment due to health=Q6/10 for all respondents.

For questions 2 to 6, the recall period is one week. For participants who missed work and did not work in the past week, the percentage of overall work impairment due to health will be the same as the percentage of work time missed due to health [14].

#### Sleep Study

We will evaluate the OSA by an overnight type III polysomnography (Nox A1, ResMed). This will involve the monitoring of several physiological signals including airflow using nasal canulae, snoring by a contact microphone placed on the anterior neck, movement of the chest and abdomen wall using respiratory inductance respiratory belts, transcutaneous arterial oxygen saturation and pulse rate by pulse oximetry as well as body position and sleep-wake pattern using actimetry. Recordings will be manually scored according to the 2012 American Association of Sleep Medicine (AASM) manual for the scoring of sleep and associated events [15]. The analysis will include the Apnea Hypopnea Index (AHI), the 3% oxygen desaturation, as well as the percentage of recording time with transcutaneous arterial oxygen saturation of <90% (T90%).

The severity of OSA will be classified as follows: non-OSA for AHI of less than 5 events per hour, mild OSA for AHI ranging from 5 to 14.9 events per hour, moderate OSA for AHI ranging from 15 to 29.9 events per hour, and severe OSA for AHI of 30 or more events per hour [16].

#### Maintenance of Wakefulness Test

The maintenance wakefulness test (MWT) is conducted throughout the day, right after the overnight full polysomnography. Its purpose is to objectively assess the ability to stay awake in drowsy conditions. The procedure will consist of 4 trials, each lasting 40 minutes with a 2-hour interval between them. It will be conducted at 8 AM, 10 AM, noon, and 2 PM using the international 10-20 system electroencephalogram electrodes F3, F4, C3, C4, O1, and O2 referred to M1 and M2, with bilateral electrooculogram and submental electromyogram, according to AASM recommendations [17].

All the trials will take place in the same bedroom, which will be soundproof and insulated from external light. The bedroom will be equipped with dim lighting, and the ambient temperature will be recorded at the beginning of each trial and maintained as close to 22 °C (72 °F) as possible. Breakfast will be provided at least an hour before the first MWT trial and lunch immediately after the noon trial. During each trial, participants will be instructed to sit up in bed with their backs and heads supported by a bedrest cushion to ensure that the neck would not uncomfortably flex or extend during any sleep episode that might occur. Their backs will be positioned at an angle of 45°-90° relative to the bed, and their legs will be straight out with some flexing at the knees to maximize comfort. Before each trial, the participants will receive the following instructions: “Please sit calmly and try to remain awake for as long as possible. Keep your gaze straight ahead and avoid looking directly at the light.” The participants will not be permitted to use any extreme measures, such as slapping their faces or singing, to stay awake. The recordings will be monitored by a trained technologist who will observe the patient via digital video and mark eye-opening and closing on the recording Each trial will end when the participant falls asleep for the first time (known as sleep onset) or after being in bed for a maximum of 40 minutes if sleep onset does not occur. Sleep onset will be defined as 3 consecutive 30-second epochs of stage 1 or any single 30-second epoch of another sleep stage (II, III, IV, or rapid eye movement). If a participant sleeps for more than 10 minutes during the first 3 trials, they will be excluded from the test. A person’s participation in the test will end after the fourth trial. MWT sleep latency will be defined as the time from trial onset to the first epoch of any sleep stage. If the participants remained awake during the whole recording session, 40 minutes were used in the calculation of the mean sleep latency (MWT, 4 trials of 40 minutes each). A mean MWT sleep latency of 19 minutes or less will indicate EDS, as suggested by the French group [18].

#### Study Procedure

Our research teams will consist of 30 voluntary occupational physicians, with at least 1 representative from each of Tunisia’s 24 provinces. Members of the research team will recruit participants at their periodic medical visits.

When eligible individuals are approached for participation, they will receive a Participant Information Sheet (PIF), and the researcher will provide an oral explanation of the project. Participants will have the opportunity to ask any questions they may have. To ensure that drivers provide accurate information regarding their work productivity and traffic accident history, participants will be assured that their responses will be kept confidential, addressing any concerns they might have about their employment. If they consent to take part, they will be asked to sign an informed consent form. Following this, they will be invited to complete the survey. The initial meeting will last approximately 30 minutes.

Individuals who display a high likelihood of OSA or EDS will be personally informed and referred to sleep centers for appropriate investigations and management. This will mainly include a sleep study and specific treatment if required.

Continuous positive airway pressure (CPAP) will be indicated for participants with severe OSA regardless of symptomatology or moderate OSA combined with ESS of 11 or greater, drowsy driving, or a history of sleep-related near misses or vehicular accidents [19].

After 1 year, all participants will be re-evaluated for their BMI, daytime sleepiness, drowsy driving, sleep-induced near misses and vehicular accidents, and work productivity. Those treated with CPAP will undergo MWT, according to the French National Authority for Health (Haute Autorité de Santé [HAS]) [20]. Adherence, tolerance, and consistency of CPAP usage will be recorded. Good compliance will be considered for those who use CPAP for at least 4 hours per night, for more than 5 days a week, and on at least 70% of nights over 30 consecutive days. We estimate the final results will be available by September 2026.

### Prediction of OSA Probability and Associated Road Traffic Accidents

#### Machine Learning Models

We will test several widely used machine learning models in medical diagnosis, known for their high accuracy. Predicting road traffic accidents using machine learning is an active area of research, with many studies leveraging various algorithms and datasets to improve accuracy and provide actionable insights. Previous studies on road traffic accident prediction have explored various machine learning algorithms. Prior studies [21-24] have shown that the best results were achieved using random forests, extreme gradient boosting, and deep neural networks. These methods have consistently demonstrated superior performance in terms of accuracy, robustness, and ability to handle complex, nonlinear relationships in traffic accident data. Random forests and extreme gradient boosting are particularly effective for structured or tabular data, offering interpretability and high predictive power, while deep neural networks excel in capturing intricate patterns in large-scale and high-dimensional datasets, especially when dealing with spatiotemporal or sequential data. Together, these methods represent the state-of-the-art in traffic accident prediction [21-24].

#### Preprocessing Data

The data will be split into 2 sets: a training set and a test set. The training set will be used to train machine learning models. The test set will be used to evaluate the models’ performance. A crucial step is planned before training the classification models, which is preprocessing. The main steps we will apply can be summarized as follows:

1. Data cleaning: handle missing values and duplicates.

2. Feature encoding: convert categorical and text data.

3. Handle outliers: detect and treat outliers.

4. Feature scaling: normalize or standardize numerical features.

5. Data splitting: divide into training, validation, and test sets.

6. Feature selection: remove irrelevant or redundant features.

### Model Evaluation

We will proceed with predicting OSA and associated road traffic accidents as a classification problem. In classification tasks, metrics, such as accuracy, precision, receiver operating characteristic area under the curve recall, and *F*_1_-score are the most relevant because they provide different perspectives on a model’s performance. Each metric highlights specific aspects of how well the model is classifying data, and their importance depends on the problem context and the cost of errors.

### Statistical Analysis

The data analysis will be conducted using SPSS Statistics for Windows, version 23.0 (IBM Corp). To determine the normal distribution of data, we will use the Kolmogorov-Smirnov test. We will report continuous variables with normal distribution as the mean and SD, while nonnormally distributed variables will be reported as the median and IQR. We will compare the means of 2 and 3 or more groups of continuous variables that are normally distributed by using the independent samples *t* test and ANOVA, respectively. The median of nonnormal continuous variables will be compared using nonparametric tests such as the Mann-Whitney *U* test and Kruskal-Wallis. Categorical variables will be presented as counts and frequencies. We will compare the frequencies of categorical variables using Pearson chi-square or Fisher exact test, when appropriate. To determine independent risk factors of OSA, EDS, drowsy driving, near misses or vehicular accidents, and impaired work productivity, we will use univariate logistic analyses. We then fit significant variables as well as those previously reported in the literature into a multivariate regression model to delineate independent factors [25]. A *P* value of <.05 will be considered significant.

### Ethical Considerations

#### Human Participants’ Ethics Review Approvals

The study received approval from the Ethics Committee of the Hedi Chaker University Hospital in Sfax, Tunisia (Reference comité éthique 04/2024, April 17, 2024). All research procedures adhered to institutional guidelines, the Declaration of Helsinki, and good clinical practice standards.

#### Informed Consent Process

Participants were given sufficient time to consider their participation in the research. To ensure voluntary participation, efforts were made to minimize the possibility of coercion or undue influence. The informed consent document was written in clear language to help participants understand the purpose, procedures, risks, benefits, and alternatives associated with the study. Consent was documented through signed forms, which were securely stored to maintain confidentiality. Participants were informed of their right to withdraw from the study at any time without facing penalties or loss of benefits. This right was clearly stated in the consent document and communicated during the consent process. No waiver of consent was requested or granted for this study. All participants provided written informed consent before their involvement. Elsewhere, this study did not include a secondary analysis of existing data.

#### Privacy and Confidentiality

While the data collected in this study were not anonymized, rigorous measures were implemented to ensure the confidentiality of participants’ information. All data were stored in a secure, password-protected environment, accessible only to authorized personnel involved in the study. Access to the data was strictly restricted to researchers directly involved in the analysis, and all personnel received training on confidentiality protocols. Electronic data were encrypted during both storage and transfer to prevent unauthorized access. Physical data storage areas were locked and monitored to deter unauthorized physical access. Participants were fully informed about the confidentiality measures during the consent process to ensure they understood how their data would be protected.

#### Compensation Details

Participants did not receive financial or material compensation for their involvement in this study. No identifiable images or data were included in the manuscript or supplementary materials.

## Results

### Sociodemographic and Health Characteristics

The study group included 127 professional drivers recruited between May and June 2024. The mean age of participants was 39.22 (SD 8.62) years, with 66.2% (83/127) of them being married. Most lived in urban areas and had completed either secondary (76/127, 60.3%) or university-level education (35/127, 27.8%). Rates of smoking and alcohol consumption were 54.3% (69/127) and 7.1% (9/127), respectively. The mean BMI was 25.6 (SD 4.22) kg/m², ranging from 15.15 to 38.87 kg/m² and 20.5% (26/127) of participants had chronic morbidity. One participant reported chronic use of hypnotics (Table 1).

**Table 1:** Sociodemographic and health characteristics of the study sample.

Variables			Study sample (N=127)
Age (years), mean (SD)			39.22 (8.62)
Male, n (%)			127 (100)
BMI, kg/m², mean (SD)			25.6 (4.22)
**Education level, n (%)**			
	Primary school	15 (11.9)	
	Secondary school	76 (60.3)	
	University-level education	35 (27.8)	
**Marital status, n (%)**			
	Single	39 (30.7)	
	Married	83 (66.2)	
	Divorced	4 (3.1)	
**Residential environment, n (%)**			
	Urban	109 (87.2)	
	Rural	16 (12.8)	
**Smoking status, n (%)**			
	Current smoker	69 (54.3)	
	Former smoker	14 (11)	
	Nonsmoker	44 (34.6)	
	Pack-years, mean (SD)	12 (10.47)	
**Chronic alcoholism, n (%)**			
	Yes	9 (7.1)	
	No	118 (92.9)	
**Chronic hypnotic use,** **n (%)**			
	Yes	1 (0.8)	
	No	126 (99.2)	
**Chronic comorbidities, n (%)**			
	Yes	26 (20.5)	
	No	101 (9.5)	

### Occupational Characteristics

Regarding occupational characteristics, the average professional seniority was 8 (SD 8.07) years, ranging from 1 to 35 years. Their average workday was 8.8 (SD 1.91) hours, and they drove a mean distance of 148.2 (SD 209.58) km daily. Machinery operators formed the largest subgroup (52/127, 41.3%), followed by truck drivers (44/127, 34.9%). Night shifts were worked by 49/127 drivers (39.5%). Among the drivers, 49/127 (39.5%) worked night shifts, and 30/127 (23.6%) were involved in transporting hazardous materials (Table 2).

**Table 2:** Occupational characteristics, sleep parameters, and work productivity of the study sample.

Variables			Study sample (N=127)
**Occupational characteristics**			
	Light vehicle, n (%)	28 (22.2)	
	Truck, n (%)	44 (34.9)	
	Semitrailer truck, n (%)	2 (1.6)	
	Machinery, n (%)	52 (41.3)	
	Seniority (years), mean (SD)	8 (8.07)	
	Hours worked per day, mean (SD)	8.84 (1.91)	
	Hours worked per week, mean (SD)	49 (9.03)	
	Distance driven per day, km, mean (SD)	148.2 (209.58)	
	Night work, n (%)	49 (39.5)	
	Transport of hazardous materials, n (%)	30 (23.6)	
**Sleep disturbances, n (%)**			
	Snoring	26 (20.6)	
	ESS^a^ score ≥ 11	24 (19.4)	
	Berlin Questionnaire positive (high Risk of OSA^b^)	13 (11.1)	
	Sleepiness while driving	33 (26.8)	
	Sleep-induced near-miss vehicle accidents	10 (7.9)	
	Sleep-induced vehicular accidents	2 (1.6)	
**WPAI: GH 2.0^c^ scores, mean (SD), %**			
	Absenteeism	1.74 (10.89)	
	Presenteeism	12.25 (24.85)	
	Productivity loss	12.76 (26.11)	
	Daily activity impairment	12.76 (23.45)	

^a^ESS: Epworth Sleepiness Scale.

^b^OSA: obstructive sleep apnea.

^c^WPAI: GH 2.0: Work Productivity and Activity Impairment Questionnaire: General Health V2.2.

### Sleep Disturbances, Work Productivity, and Activity Impairment

Almost one-fifth of the participants reported habitual snoring, and more than 1 quarter reported experiencing sleepiness while driving. Based on the ESS, 19.4% (24/127) of the drivers scored 11 or higher, indicating EDS. The Berlin Questionnaire classified 11.1% (n/N) as having a high risk for OSA. Regarding driving safety outcomes, 7.9% (10/127) reported at least one near-miss vehicle accident, while 1.6% (2/127) reported involvement in a vehicle crash. A total of 30 cases (30/127, 23.62%) were identified with either a pathological ESS (ESS ≥11) or a positive Berlin score, all these individuals were referred to specialized sleep centers for further evaluation and therapeutic management.

Professional drivers exhibited considerable variation in work productivity and activity impairment (Table 2). The mean score for absenteeism was 1.74% (SD 10.89%), while presenteeism had a higher mean score of 12.25% (SD 24.85%). The mean score for total productivity loss was 12.76% (SD 26.11%), indicating that both personal and work-related issues significantly affect overall productivity. Daily activity impairment, with a mean score of 12.76% (SD 23.45%), reflected the overall productivity loss.

## Discussion

### Strength of the Study

This cohort study has the goal of providing accurate information regarding the prevalence of sleep-wake disturbance among professional drivers, and how it affects work productivity and road safety. The study also aims to develop a simple and reliable screening tool for OSA among professional drivers and to improve treatment practices. It will be the first large-scale study of its kind in Tunisia, a country with a high rate of road accidents. Professional drivers are at high risk of OSA, which is often going undiagnosed or untreated, and it may contribute significantly to these accidents. Currently, there are no clear regulations in Tunisia regarding fitness-to-drive for individuals with sleep-wake disturbances, and physicians may not be sufficiently aware of these issues.

The study will use a range of validated self-reported measures in the local language to investigate sleep-wake status and work impairment, which will improve response rates and data accuracy. Participants who are deemed to be at high risk of OSA or have EDS will be referred to sleep centers for investigations which may help obtain a more accurate estimation of the OSA prevalence among professional drivers. Those diagnosed with moderate to severe OSA with EDS will be treated with CPAP and re-evaluated after 1 year of follow-up with self-report questionnaires and MWT. This will allow us to assess the impact of CPAP on daytime wakefulness and work productivity.

The study results will be shared with internal and external stakeholders to raise awareness about this issue and improve screening and treatment practices. It would also have a significant impact on legal regulations regarding fitness-to-drive in sleep-wake disturbances, which would improve road safety in the country. The study team believes that road safety should be a priority, and they hope that the results will help achieve this objective. The demonstrated cost savings and crash reduction reinforce the importance of standardized screening protocols, a point we emphasize in discussing policy recommendations for Tunisian transport sectors [26].

### Limitations of the Study

This study has several limitations that should be considered. First, similar to the findings of Burks et al [26], we recognize that fear of job loss or related stigma may prevent drivers from disclosing their symptoms of obstructive sleep apnea. This underscores the importance of using anonymized data collection methods, collaborating with third-party health organizations, and implementing legal protections to encourage individuals to disclose their health issues without fear of negative consequences. In addition, using police reports to cross-reference accident data with objective crash records can help mitigate biases associated with self-reporting.

Second, the small number of women in our sample makes it difficult to ensure that our findings accurately reflect the prevalence of OSA and EDS among female professional drivers. To address this, we will analyze the data separately by gender to gain a clearer understanding of the situation. In addition, our method for screening snoring relies solely on self-reports from participants, which may underestimate its prevalence since some individuals may be unaware of their snoring, especially if they sleep alone. Finally, while type 1 polysomnography is considered the gold standard for diagnosing OSA, our financial constraints limit us to conducting only type 3 overnight polysomnography in this study, which may also lead to an underestimation of OSA prevalence.

### Conclusion

This study is the first of its kind in Tunisia, aiming to investigate the prevalence of OSA, EDS, and work productivity impairment among professional drivers. The main objective of this research is to raise awareness among physicians about sleep and wake disturbances among drivers and to improve OSA screening and treatment practices. Furthermore, we plan to develop a simple algorithm for screening OSA during prerecruitment examinations or periodic visits. We also hope that the study's findings will help establish legal guidelines in Tunisia regarding the fitness-to-drive of individuals with sleep-wake disturbances. We firmly believe that ensuring road safety should become a political priority in cities, and we hope that our research will be beneficial in achieving this objective in our country.

## Appendix

Multimedia Appendix 1 English version of the Berlin questionnaire.

Multimedia Appendix 2 English version of the Epworth Sleepiness Scale (ESS).

Multimedia Appendix 3 English version of the Work Productivity and Activity Impairment Questionnaire: General Health V2.2 (WPAI: GH 2.0).

## Abbreviations

AASM: American Association of Sleep Medicine
AHI: apnea hypopnea index
CPAP: continuous positive airway pressure
EDS: excessive daytime sleepiness
ESS: Epworth Sleepiness Scale
HAS: Haute Autorité de Santé
MWT: Maintenance of Wakefulness Test
OSA: obstructive sleep apnea
PIF: participant information sheet
WPAI: GH 2.0: Work Productivity and Activity Impairment Questionnaire: General Health V2.2
