# Supplementary figures and images for: Screening and Management of Obstructive Sleep Apnea and Daytime Sleepiness Among Professional Drivers in Tunisia: Protocol for a Machine Learning Study
Source: JMIR Res Protoc. 2025 Aug 15;14:e70441. doi: 10.2196/70441 (PMC12397752; doi:10.2196/70441)

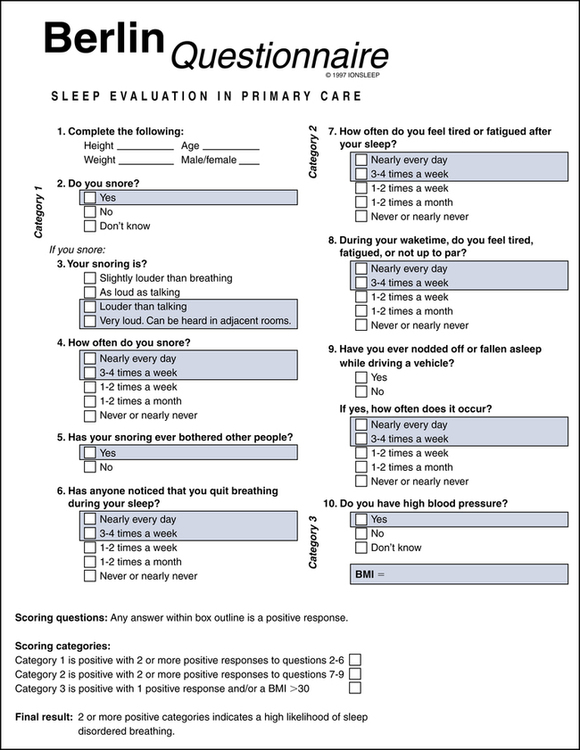


**Multimedia Appendix 1**: English version of the Berlin questionnaire

Supplement: Multimedia Appendix 1 [file resprot_v14i1e70441_app1.docx]

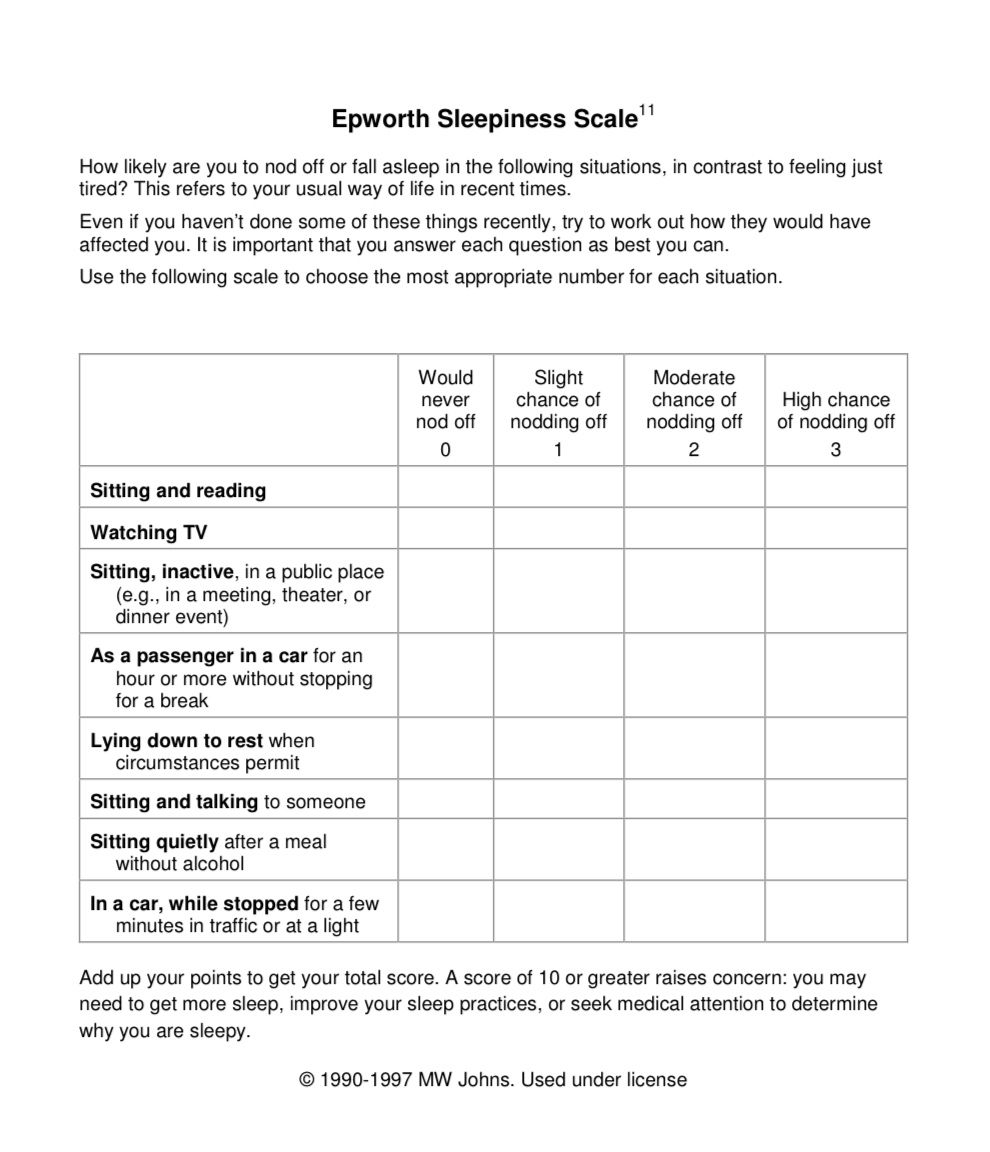


**Multimedia Appendix 2**: English version of the Epworth Sleepiness Scale (ESS)

Supplement: Multimedia Appendix 2 [file resprot_v14i1e70441_app2.docx]
