# Supplementary material for: Screening and Management of Obstructive Sleep Apnea and Daytime Sleepiness Among Professional Drivers in Tunisia: Protocol for a Machine Learning Study
Source: JMIR Res Protoc. 2025 Aug 15;14:e70441. doi: 10.2196/70441 (PMC12397752; doi:10.2196/70441)
Supplement: Multimedia Appendix 3 [file resprot_v14i1e70441_app3.docx]

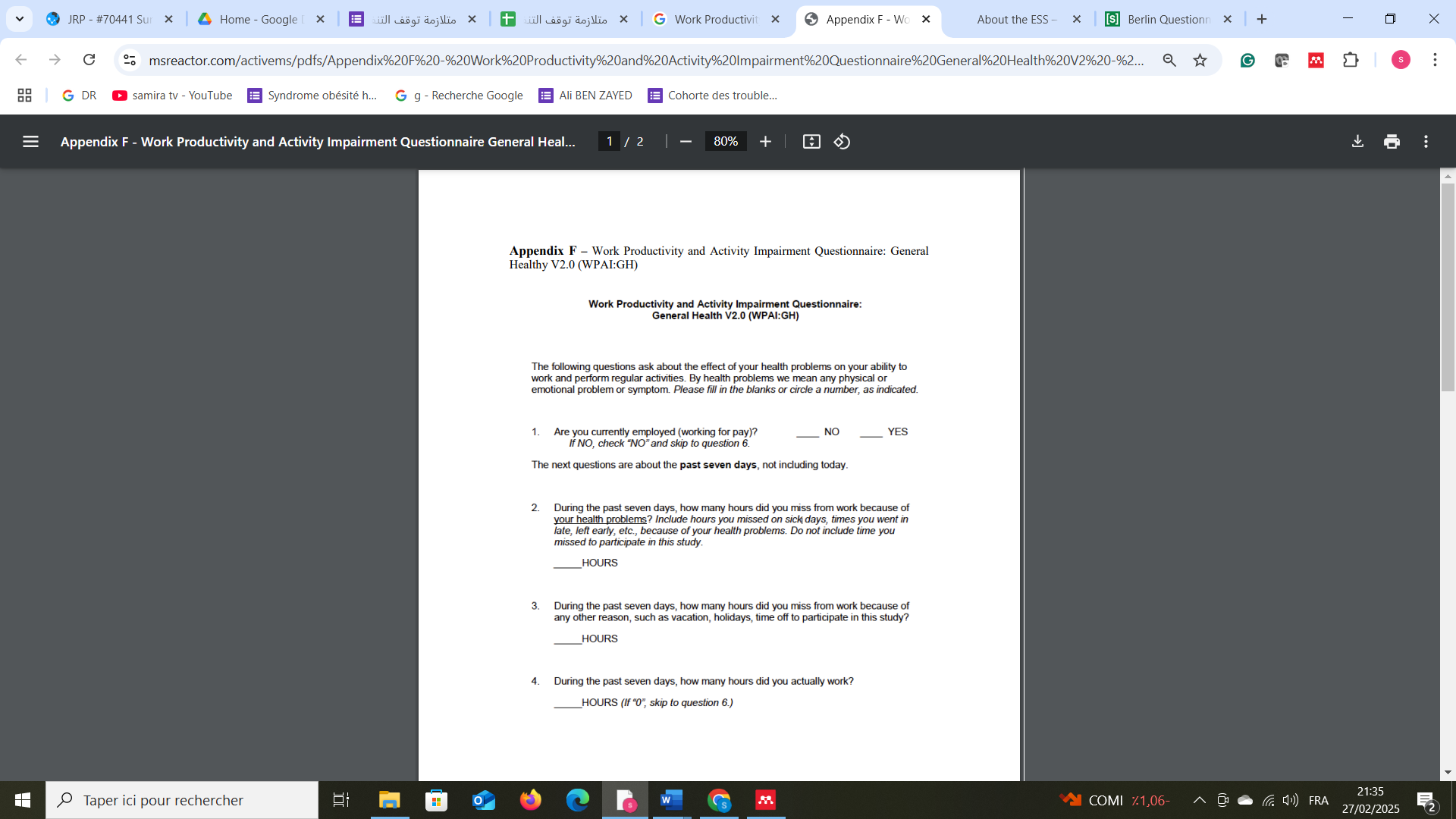


**Multimedia Appendix 3**: English version of the Work Productivity and Activity Impairment Questionnaire: General Health V2.2 (WPAI: GH 2.0)
